# The Role of Aldosterone in OSA and OSA-Related Hypertension

**DOI:** 10.3389/fendo.2021.801689

**Published:** 2022-01-12

**Authors:** Yi Wang, Chuan Xiang Li, Ying Ni Lin, Li Yue Zhang, Shi Qi Li, Liu Zhang, Ya Ru Yan, Fang Ying Lu, Ning Li, Qing Yun Li

**Affiliations:** ^1^ Department of Pulmonary and Critical Care Medicine, Ruijin Hospital, Shanghai Jiao Tong University School of Medicine, Shanghai, China; ^2^ Institute of Respiratory Diseases, Shanghai Jiao Tong University School of Medicine, Shanghai, China; ^3^ Shanghai Key Laboratory of Emergency Prevention, Diagnosis and Treatment of Respiratory Infectious Diseases, Ruijin Hospital, Shanghai, China; ^4^ Department of Respiratory and Critical Care Medicine, Tongren Hospital Affiliated to Wuhan University, The Third Hospital of Wuhan, Wuhan, China

**Keywords:** obstructive sleep apnea, aldosterone, hypertension, continuous positive airway pressure (CPAP), mineralocorticoid receptor antagonists

## Abstract

Obstructive sleep apnea (OSA) is regarded as an independent risk factor for hypertension. The possible mechanism includes oxidative stress, endothelial injury, sympathetic excitement, renin–angiotensin–aldosterone system activation, etc. Clinical studies have found that there is a high coexistence of OSA and primary aldosteronism in patients with hypertension and that elevated aldosterone levels are independently associated with OSA severity in resistant hypertension. The underlying mechanism is that aldosterone excess can exacerbate OSA through increasing overnight fluid shift and affecting the mass and function of upper airway muscles during the sleep period. Thus, a bidirectional influence between OSA and aldosterone exists and contributes to hypertension in OSA patients, especially resistant hypertension.

## Introduction

Obstructive sleep apnea (OSA) is characterized by repetitive overnight hypoxic episodes and subsequent sleep fragmentation due to a complete or partial collapse of the upper airway. It was estimated that 936 million adults aged 30–69 years have OSA globally, including 425 million moderate to severe ones (1). OSA is related to not only endothelial injury and sympathetic excitement but also endocrine dysfunction such as elevated secretion of catecholamine and renin–angiotensin–aldosterone system activation (2–5). Those mechanisms make OSA a well-established independent risk factor of HTN (6). About 25% hypertensive patients had OSA (7). The incidence of OSA could reach 83%~92.3% among patients with resistant hypertension (RHTN) (8, 9). Untreated moderate to severe OSA increased the risk of RHTN (OR = 2) (10). Recently, it has been noticed that OSA is highly present in primary aldosteronism (PA) and vice versa. Meanwhile, elevated aldosterone is associated with OSA severity in RHTN. Herein, we will give a review of the role of aldosterone in OSA and its related HTN.

## Coexistence of PA and OSA

### OSA in PA

As we know, OSA brings great health burden in the general population. Higher incidence of OSA in PA patients was identified (**Table 1**). The RESIST-POL study found that OSA occurred more frequently in RHTN patients with PA than those without PA (59.4% vs. 42.4%) (15). In a population consisting of 3,003 consecutive patients with HTN, 321 were diagnosed with PA, among whom 45.8% were OSA patients (14). Then, a cross-sectional multiethnic study that screened for OSA in 207 patients with PA showed that the prevalence was 67.6% and there was no obvious difference of the prevalence between the white and Chinese (64.4% vs. 70%) (13). It was also reported the OSA prevalence of 55% among 71 Japanese patients with PA. Body mass index (BMI) and triglyceride content increased the risk of OSA in PA patients (OR = 1.27 and OR = 1.01, respectively) (11). Besides, an early retrospective study reported that among 3,428 hypertensive patients, patients with hyperaldosteronism had a higher prevalence of sleep apnea than those without hyperaldosteronism (18% vs. 9%), despite a lack of confirmation of PA (16). It should be mentioned that a much lower prevalence of OSA (10%) in a retrospective study including 677 PA patients may be attributed to the fact that polysomnography (PSG) was only conducted in those with snoring, which resulted in the underestimation of the actual prevalence of OSA in PA (12).

**Table 1:** The prevalence of OSA in PA patients.

Author	Study design	Nation	Subjects	Assessment of PA	Assessment of OSA	Prevalence of OSA
Nakamura (11)	Cross-sectional	Japan	71 PA	Screening test (ARR > 20 ng/dl: ng/ml/h) and confirmatory test including CCT (ARR ≥ 20), FUT (PRA < 2.0 ng/ml/h), and SIT (aldosterone level ≥ 6.0 ng/dl). PA was diagnosed if ≥1 of the 3 tests were positive.	Smart Watch PMP-300E (REI ≥ 15 or REI ≥ 5 together with symptoms)	55%
Li (12)	Retrospective	China	677 PA	Screening test (ARR > 24 ng/dl: ng/ml/h) and confirmatory test (SIT: aldosterone level ≥ 6.0 ng/dl)	PSG (used only in snoring patients with PA) (AHI ≥ 5)	10%
Buffolo (13)	Cross-sectional, multiethnic, multicenter	Europe	104 PA	PA was diagnosed according to the 2016 ES guideline. Different centers have different standards for the screening test and confirmatory test.	Cardiorespiratory polygraphy (AHI ≥ 5)	64.40%
		China	100 PA	Same as above	Same as above	70%
Wang (14)	Cross-sectional study	China	321 PA	A suppressed PRA (<1.0 μg/L/h) and an elevated aldosterone level (>12 ng/L) or an elevated ARR (>20 ng/dl: ng/ml/h) and a confirmation test by saline infusion test (aldosterone level was >5 ng/dl)	All patients suspicious of OSA underwent full-night PSG (AHI ≥5)	45.80%
Prejbisz (15)	Cross-sectional	Poland	32 PA and 172 non-PA amid 204 RHTN	screening test (ARR > 30 ng/dl: ng/ml/h, PAC > 15 ng/dl) and confirming test (CCT: aldosterone level fails to decrease by more than 30%)	All patients irrespective of the symptoms of OSA were evaluated by PSG (AHI ≥ 15)	59.4% in PA; 42.4% in non-PA, *P* = 0.058
Sim (16)^a^	Retrospective	USA	3,428 HTN (575 had HA	HA was defined as ARR >30 (ng/dl: ng/ml/h) and PAC >20 ng/dl or ARR >50	Sleep apnea was defined by ICD-9 coding or procedural coding for dispensation of positive airway devices.	18% in HA vs. 9% in non-HA (P<0.001)

OSA, obstructive sleep apnea; HTN, hypertension; RHTN, resistant hypertension; PA, primary aldosteronism; HA, hyperaldosteronism; ESS, Epworth Sleepiness Scale; PSG, polysomnography; AHI, apnea–hypopnea index; PAC, plasma aldosterone concentration; PRA, plasma renin activity; ARR, aldosterone-to-renin ratio; UAldo, urine aldosterone level; CCT, the captopril challenge test; FUT, the furosemide upright test; SIT, the saline infusion test; ICD-9, International Classification of Disease, Ninth Revision.

a This retrospective study did not have PA confirmed.

Management of PA improved OSA symptoms. Wolley et al. performed repeated PSG on 20 patients with PA pre- and post-treatment. Seven patients underwent adrenalectomy and 13 patients received treatment with spironolactone, amiloride, or both. The mean apnea–hypopnea index (AHI) was reduced from 22.5 to 12.3 events/h (*P* = 0.02) after an 8-month follow-up (17). A retrospective study including 83 PA patients (48 surgically managed and 35 medically managed) also showed a significant reduced OSA probability assessed by the Berlin questionnaire after PA treatment (from 64% to 35%, mean Berlin score 1.64 to 1.35, *P* < 0.001) with the follow-up duration up to 2.6 years. It seemed to be independent of BMI, for BMI remains unchanged before and after treatment for PA. Both obese and non-obese patients showed significant decreases in OSA probability (18).

### PA in OSA

PA was present in 2.6%–12.7% of the general HTN patients (19). Studies showed that the incidence of PA in OSA patients was at least 5% in OSA-HTN patients and up to 36% in RHTN patients with probable OSA (13, 14, 20–23) (**Table 2**). PA is likely to be more frequent in patients with OSA and HTN than in the general hypertensive population. Among 114 subjects with RHTN, 72 subjects had a high risk for sleep apnea based on the Berlin questionnaire who were more likely to have a diagnosis of PA than those with low risk of sleep apnea (36% vs. 19%) (20). Furthermore, the study of Di Murro et al., in which both PA and OSA were definitely confirmed, found that OSA patients (*n* = 53) had a higher prevalence of PA compared with non-OSA patients (*n* = 272) (34% vs. 9.2%) (21).

**Table 2:** The prevalence of PA among OSA.

Author	Study design	Nation	Subjects	Assessment of PA	Assessment of OSA	Prevalence of PA
Dobrowolski (23)	Prospective	Poland	94 moderate/severe OSA and HTN	Confirmed by SIT (aldosterone level > 10 ng/dl) plus low baseline PRA	PSG (AHI ≥ 15)	21.30%
Chee (22)^a^	Prospective	Australia	40 OSA and HTN	Only screening test performed [ARR > 70 pmol/mU (approximate 20 ng/dl: ng/ml/h)]	PSG (AHI ≥ 5)	5% had likely PA and 22.5% had possible PA
Buffolo (13)	Cross-sectional, multiethnic, multicenter	Europe	102 patients with OSA and HTN	PA was diagnosed according to the 2016 ES guideline. Different centers have different standards for the screening test and confirmatory test	Cardiorespiratory polygraphy (AHI ≥ 5)	11.80%
		China	101 patients with OSA and HTN	Same as above	Same as above	5.90%
Wang (14)	Cross-sectional study	China	888 patients with OSA and HTN	A suppressed PRA (<1.0 μg/L/h) and an elevated aldosterone level (>12 ng/L) or an elevated ARR (>20 ng/dl: ng/ml/h) and a confirmation test by SIT (aldosterone level was >5 ng/dl)	PSG (AHI ≥ 5)	16.55%
Di Murro (21)	Prospective	Italy	53 OSA and HTN	ARR >40 (ng/dl: ng/ml/h) in the presence of PAC >15 ng/dl and suppressed PRA and confirmatory test [SIT (PAC >5 ng/dl)]	Only those have features of OSA and ESS ≥10 underwent PSG (AHI ≥ 5)	34%
Calhoun (20)	Prospective	USA	114 RHTN (72 subjects had a high probability and 42 subjects had a low probability of having sleep apnea)	PA was defined as a suppressed PRA (<1.0 ng/mL/h) and elevated 24-h UAldo >12 μg in the setting of high dietary sodium ingestion (>200 mEq/24 h)	Berlin questionnaire	Subjects at high risk for sleep apnea were almost two times more likely to have PA diagnosed (36% vs. 19%, *P* < 0.05)

OSA, obstructive sleep apnea; HTN, hypertension; RHTN, resistant hypertension; PA, primary aldosteronism; PSG, polysomnography; ESS, Epworth Sleepiness Scale; AHI, apnea–hypopnea index; PAC, plasma aldosterone concentration; PRA, plasma renin activity; ARR, aldosterone-to-renin ratio; UAldo, urine aldosterone level; SIT, the saline infusion test.

a This study inferred that the likelihood and possibility of PA in hypertensive OSA patients were 5% and 22.5% mainly based on the results of screening test and usage of antihypertensive agents, because no one completed the confirmation test.

There was a high coexistence of PA and OSA, discrepancies in the results notwithstanding. The possible reason for the variation in prevalence should be considered such as sample sizes, differences in the criteria for the selection of patients, and different methodologies and criteria for the diagnosis of PA and OSA (**Tables 1** and **2**).

## Aldosterone Level in OSA

### Higher Aldosterone Levels in OSA

Several studies showed that aldosterone excess was present in OSA and suggested its involvement in the pathogenesis of OSA-related HTN. A meta-analysis showed that OSA patients tended to have a higher aldosterone level compared with the control group. A subgroup analysis further found that this effect was solely significant in OSA patients with HTN rather than in OSA patients without HTN (24). Later, a cross-sectional study with 534 primary RHTN patients (493 OSA and 41 non-OSA) demonstrated that the severe OSA group had elevated plasma aldosterone concentration (PAC) compared with those in the non-OSA group or mild/moderate OSA group. PAC and 24-h urine aldosterone level (24-h UAldo) were positively associated with AHI (*β* = 0.32 and *β* = 0.35, respectively, *P* < 0.05), after adjustment of age, gender, BMI, smoking status, plasma renin activity (PRA), diuretic and angiotensin-converting enzyme inhibitors (ACEI), and/or angiotensin receptor blocker (ARB) usage (9). Similarly, de Souza and colleagues found that higher PAC and 24-h UAldo were associated with severe OSA after adjustment for age, sex, BMI, 24 h BPs, and dipping status (25).

Studies have explored the impact of continuous positive airway pressure (CPAP), the gold-standard therapy for moderate to severe OSA, on aldosterone levels in OSA patients with a follow-up varying from 1 to 14 months (25–34) (**Table 3**). Concerning about normotensive population, two observational studies were conducted by Nicholl and colleagues to evaluate the effect of 1-month CPAP on renin–angiotensin–aldosterone system (RAAS) activity in OSA patients without HTN and diabetes in 2014 and 2021. Not only PAC was significantly reduced but also BP and renal function were improved after CPAP treatment (26, 28), especially in those with severe hypoxemia (26). Of note, patients in both studies were asked to keep a high-salt diet 3 days before the first visit until the second visit to avoid the impact of salt status on the aldosterone level. However, Møller and coworkers found that no laboratory data including PAC were changed in 13 symptomatic OSA patients without HTN after a successful 14-month CPAP treatment (29). A parallel randomized controlled trial (RCT) of 1-month CPAP enrolling 101 males with OSA was conducted, in which both the placebo and active treatment groups have similar use of CPAP. Unexpectedly, PAC rose by 30% in both groups and the sham/active difference was not significant (33). The result should be interpreted with caution due to lack of demographic information about coexisting diseases and drug usages.

**Table 3:** Effect of CPAP on aldosterone levels.

Author	Nation	Study design	Number (male)	With HTN?	Follow-up (months)	Compliance (h/night)	Outcome
Nicholl (26)	Canada	Observational	30 (20)	Normotensive	1	>4	PAC ↓
Nicholl (28)	Canada	Observational	20 (15)	Normotensive	1	>4	PAC ↓
Møller (29)	Denmark	Observational	13 (12)	Normotensive	14	Sufficient compliance	PAC, PRA, Ang II →
Meston (33)	Britain	RCT	101 (101)	No data	1	Placebo: 4.6 ± 2.4; active: 5.4 ± 1.6	PAC ↑ in both groups and sham/active differences →
Joyeux-Faure (34)	Spain	RCT	37 (32)	RHTN	3	CPAP: 3.90; sham CPAP: 1.86	Increase of PAC was significant in the sham CPAP group compared with active CPAP; renin →
De Souza (25)	Brazil	RCT	117 (47)	RHTN	6	>4 (45 patients in the CPAP group meet good compliance)	24-h UAldo
							↓ solely in patients with true RHTN, but not in those with whitecoat RHTN
Sánchez-de-la-Torre (27)	Spain	Observational	37 (37)	RHTN	3	>4	PAC →
							Decrease of ARR was significantly greater in the responder group (*n* = 18)
Lloberes (32)	Spain	RCT	78 (59)	RHTN	3	5.6 ± 1.5	PAC ↓ was found in nine patients with whitecoat RHTN, but not in the 27 patients with true RHTN
Pedrosa (31)	Brazil	Randomized	35 (27)	RHTN	6	6.01 ± 0.20	PAC →
Saarelainen (30)	Finland	Observational	11 (11)	HTN	3	>4	PAC ↓, renin →

OSA, obstructive sleep apnea; HTN, hypertension; RHTN, resistant hypertension; AHI, apnea–hypopnea index; BP, blood pressure; PAC, plasma aldosterone concentration; PRA, plasma renin activity; CPAP, continuous positive airway pressure; RCT, randomized controlled trial; ARR, aldosterone-to-renin ratio; UAldo, urine aldosterone; Ang Ⅱ, angiotensin Ⅱ; ↑, significantly increase; ↓, significantly decrease; →, insignificant change.

The change of aldosterone level by CPAP treatment was also tested in OSA patients with HTN or RHTN. PAC in 11 hypertensive OSA subjects without any medications was found to be significantly decreased after 3 months of CPAP treatment (*P* = 0.046) (30). Improvement in nighttime mean BP was associated with changes of aldosterone. Lloberes et al. conducted one RCT, which showed that a 3-month CPAP treatment significantly decreased PAC and this change was independently associated with changes in office DBP (32). Intriguingly, Joyeaux-Faure and colleagues reported a significant increase in PAC during a 3-month sham CPAP compared with the active CPAP (*P* = 0.01) (34). The 24-h UAldo tended to decrease after 6 months of CPAP treatment in another RCT (*P* = 0.074) (25). This reduction was more noticeable in those with the non-dipping pattern, not using spironolactone, less obese, and with lowest sleep SaO_2_ levels (25). Although some studies found unchanged PAC by CPAP (27, 31), a significant decrease of aldosterone-to-renin ratio (ARR) was observed in the responder group whose mean blood pressure (BP) was reduced by at least 4.5 mmHg after CPAP treatment (27).

In summary, a higher level of aldosterone was present in hypertensive OSA patients, and most studies indicated a decline of aldosterone level after CPAP treatment. Several factors blamed for some controversy in the results may belong to methodological concerns such as different demographic characteristics (OSA severity, BMI) and inconsistent control of variables (antihypertensive drugs, salt status, body position when sampling, etc.) known to influence aldosterone production.

### Possible Mechanism

Aldosterone is a hormone mainly secreted by the adrenal cortex that regulates electrolyte and water balance by increasing the renal retention of sodium and the excretion of potassium. Angiotensin II (Ang II) binds to its receptor (AT1R) on the zona glomerulosa cells of the adrenal cortex, which is one of major regulators of aldosterone synthesis. Chronic intermittent hypoxia (CIH)-induced sympathetic outflow to the kidney stimulates renin release and leads to elevated circulating levels of Ang II (35). It was demonstrated that Ang II was much higher in OSA patients than in healthy controls (24). CIH-treated rats also caused increasing levels of circulating renin activity and Ang II (36). *In-vitro* studies also confirmed that CIH directly upregulated the expression of renin (37). Besides, obesity, highly concomitant in OSA, has been found to participate in aldosterone excess in RHTN patients (38). Adipocyte-derived factors may be involved in adrenal aldosterone synthesis. Leptin was able to directly activate aldosterone synthase (CYP11B2) of the adrenal gland, resulting in increased production of aldosterone *via* a Ca^2+^-dependent mechanism which is independent of RAAS and sympathetic nervous systems (39). However, a subsequent experiment found the effect of leptin on aldosterone synthesis only happened in female mice (40). It was also observed that complement-C1q TNF-related protein-1 (CTRP-1), a secreted protein derived from adipocytes, could also enhance aldosterone production in cells of the human adrenal cortical cell line (41). Additionally, adipose tissue could secrete aldosterone itself (42). Nevertheless, studies are needed to clarify this mechanism in OSA.

## Aldosterone Excess Exacerbates OSA

### Higher Aldosterone Level Contributes to OSA

In a 109 RHTN population, the severity of OSA was found to be greater in the high PAC (defined as PRA < 1 ng/ml/h and 24-h UAldo ≥ 12 µg) group than in the normal PAC group (AHI, 19.9 vs. 10.0 events/h). PAC and 24-h UAldo were positively and significantly correlated with AHI in the high PAC group, which was not found in the normal PAC group (43). Moreover, hyperaldosteronism [defined as ARR > 30 and PAC > 20 ng/dl or ARR > 50 (ng/dl: ng/ml/h)] was reported to independently increase the risk of OSA after adjustment of age, sex, BMI categories, diabetes mellitus, and chronic heart failure (OR = 1.8, 95% CI: 1.3–2.6) (16).

Two main mineralocorticoid receptor (MR) antagonists, spironolactone and eplerenone, are currently used as secondary agents in hypertensive patients (6). Several studies evaluated their effectiveness on OSA combined with uncontrolled HTN or RHTN (44–48) (**Table 4**).

**Table 4:** Effect of aldosterone inhibition on OSA.

Author	Nation	Study design	Subjects	Intervention	Follow-up (months)	Outcome
Krasińska (48)	Poland	RCT	102 RHTN and OSA patients (*n* = 51 per group)	Therapy group: additional use of eplerenone (50 mg/daily)	6	Nighttime BP parameters, left ventricular hypertrophy, AHI, PAC ↓
				Control group: standard antihypertensive agents		
Yang (44)	China	RCT	30 RHTN and OSA patients (*n* = 15 per group)	Therapy group: additional spironolactone 20 mg once daily or 40 mg once their BP remains uncontrolled at 4 weeks	3	AHI, BP, and PAC ↓
				Control group: usual antihypertensive agents		
Krasińska (45)	Poland	Observational	31 RHTN and OSA patients	Eplerenone at a dose of 50 mg/day with a standard antihypertensive therapy	3	AHI, neck circumference, BP, aortic pulse wave, and arterial wall stiffness ↓
Kasai (46)	Canada	Observational	16 OSA patients with uncontrolled HTN	Intensified diuretic therapy (metolazone 2.5 mg and spironolactone 25 mg daily for 7 days after which the daily dose was doubled for 7 additional days)	2 weeks	AHI, BP, overnight change in leg fluid volume and overnight change in neck circumference ↓
Gaddam (47)	America	Observational	12 RHTN and OSA patients	Additional therapy (spironolactone 25 mg once daily and force-titrated to 50 mg once daily at 4 weeks)	2	Body weight, BP, AHI ↓ and PRA ↑, a tended but insignificant reduction neck circumference

OSA, obstructive sleep apnea; HTN, hypertension; RHTN, resistant hypertension; AHI, apnea–hypopnea index; BP, blood pressure; PAC, plasma aldosterone concentration; PRA, plasma renin activity; RCT, randomized controlled trial; ↑, significantly increase; ↓, significantly decrease; →, insignificant change.

Gaddam and coworkers recruited 12 individuals with OSA and RHTN, who administered additional spironolactone. All subjects were asked to remain on their baseline thiazide diuretic, ACEI, and ARB throughout the treatment period. After an 8-week therapy, the reduction in AHI was significant (39.8 ± 19.5 vs. 22.0 ± 6.8 events/h), with a significant decrease of body weight, clinic systolic and diastolic BP, and 24-h ambulatory blood pressure monitoring (AMBP) parameters (24-h systolic BP, for instance, 147 ± 13 vs. 130 ± 19 mmHg) (47). Thirty patients with OSA and RHTN were randomly assigned to a control group who received usual antihypertensive agents and a treatment group who received add-on therapy of spironolactone. After a 12-week follow-up, the reductions of clinic BP, AMBP parameters (mean differences of 24-h systolic BP in the two groups, 16.3 ± 10.0 vs. 5.3 ± 7.0 mmHg), and AHI (mean differences in the two groups, 21.8 ± 15.7 vs. 1.8 ± 12.8 events/h) were significant, as well as PAC (44). Similar results were also observed in 16 OSA patients with uncontrolled HTN. They took metolazone and spironolactone daily for 1 week after which the daily dose was force-titrated to double dose for another week. Then, a significant reduction of AHI, edema scale, body weight, total body fluid and leg fluid volume, and home BP parameters was observed (46).

Krasińska and coworkers led a 3-month observational study and a 6-month RCT in 2014 and 2019, respectively, in which additional use of eplerenone had similar benefits (significant reductions in the AHI, neck circumference, BP), along with improvement in aortic pulse wave, arterial wall stiffness, and left ventricular hypertrophy (45, 48).

The present observational and randomized trials consistently reported obvious improvement of AHI and BP after an add-on therapy of eplerenone or spironolactone in OSA patients with RHTN.

### Possible Mechanisms

Aldosterone promotes sodium–water reabsorption and elevates overnight fluid shifting to the neck in the supine position, resulting in pharyngeal edema and upper airway obstruction. It was proven that OSA severity was strongly correlated with an overnight reduction of leg fluid volume and of calf circumference (49). Clinical studies showed that aldosterone blockade not only reduces PAC and BP but also AHI and neck girth significantly (44, 45, 47, 50). Aldosterone excess could damage the taste sensitivity for sodium chloride (NaCl) and favor more salt intake, further facilitating water–sodium retention (51). Besides, significantly increased secretion of cortisol in patients with PA, independent of PA subtype or adenoma tissue genotypes, could also increase risk of OSA (52, 53).

**Figure 1:**
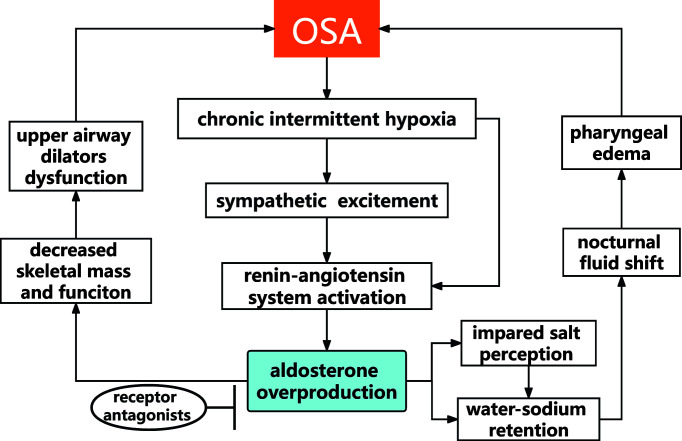
The possible pathophysiological link between OSA and aldosterone.

Aldosterone, which was related to sarcopenia (54), might affect the mass and function of upper dilator muscles. Lower skeletal muscle mass was found in female patients with PA than those with non-functional adrenal incidentaloma (55). It was also found that aldosterone impeded myogenesis *in vitro* and *in vivo* (56, 57). Both undifferentiated myoblasts and differentiated myotubes express aldosterone receptors (58). In Duchenne muscular dystrophy (DMD) mice model, the non-steroidal mineralocorticoid receptor antagonist (finerenone) brought significant improvements in clinically relevant functional parameters in skeletal muscle (normalized grip strength, lower susceptibility to limb muscle damage, normalized limb muscle force) (59) (**Figure 1**).

## Discussion and Future Perspectives

OSA is a long-lasting condition with early repetitive oxidative stress injury and proinflammatory releases, leading to systemic and local inflammation, sympathetic nervous system excitement, renin–angiotensin system activation, and aldosterone overproduction. The consequences are endothelial dysfunction, artery constriction, arterial stiffness, and water–sodium retention, which are potent pathogenic factors for HTN.

In this review, we highlighted the role of aldosterone in OSA-related HTN. Hyperaldosteronism existed in OSA and in turn aggravated OSA, which could set off the onset and development of RHTN. Moreover, with recent studies revealing a proinflammatory, profibrotic, and proinsulin resistance role of MR activation, which could predispose OSA to cardiovascular and metabolic abnormality, it is vital to consider the benefits of PA screening and aldosterone blockade during the course of treatment for patients with OSA and HTN (60).

Although the current recommendation of the Endocrine Society guideline is that all OSA patients should be screened for PA, irrespective of the grade of HTN (61), the screening rate (2.1% in persons with RHTN and 3% in hypertensive OSA inpatients) for PA remained much lower than its prevalence (62, 63). According to the latest decision analysis by weighing the cost of the indiscriminate screening test for PA in all hypertensive OSA patients and of the cardiovascular sequelae from PA when it remains unscreened, Chomsky-Higgins and colleagues indicated that screening per guideline recommendations is cost-saving which had a lower expected cost and higher quality adjusted life-years compared with the current rates of screening (64). Thus, there is great scope to enhance the screening rate of PA. It should be mentioned that aldosterone is a steroid with a low concentration in plasma and is difficult to measure with radioimmunoassay, causing widely varied PAC values across laboratories. Moreover, most studies in this review measured PAC by radioimmunoassay, which might bring some bias for the results. Thus, more accurate and rapid detection methods such as liquid chromatography–tandem mass spectrometry technique and automated chemiluminescent assays are needed to be promoted (65).

Concerning the treatment for hypertensive patients with OSA, most patients would be recommended a CPAP therapy. However, the improvement of BP control by CPAP was relatively modest (2 mmHg for patients with HTN and 5 mmHg for RHTN) (66, 67). What is worse, incompliance to CPAP is high which do affect its effectiveness (68, 69). Moreover, five studies consistently reported that MR antagonists could improve both BP control and OSA severity. Therefore, taking aldosterone blockade as complementary therapy in patients with OSA and RHTN is likely to be a useful strategy. However, there are still some questions that remain to be answered. Should MR antagonists be prioritized to be prescribed if deemed well tolerated in OSA patients with RHTN before considering CPAP? Although its application in the treatment of HTN is still in its infancy, can finerenone with higher tolerance and more safety advantages offer a better alternative? Is there a protective role of the MR antagonists on cardiovascular outcome in general hypertensive patients with OSA?

## Conclusion

Current clinical research supports a bidirectional influence between aldosterone level and OSA. More studies are needed to elucidate the underlying pathophysiological link between OSA and aldosterone. Aldosterone blockade is an effective adjunctive therapy for both OSA and OSA-related RHTN.

## Author Contributions

Conception or design of the work: YW, CL, YL, and QL. Drafting the work: YW, CL, YL, and QL. Revising the work critically for important intellectual content: YW, CL, YL, LYZ, SL, LZ, FL, YY, NL, and QL. Final approval of the version submitted for publication: YW, CL, YL, LYZ, SL, LZ, FL, YY, NL, and QL.

## Funding

This work was supported by grants from the National Natural Science Foundation of China (82070089, 81770084); Shanghai Municipal Key Clinical Specialty (shslczdzk02202); Shanghai Top-Priority Clinical Key Disciplines Construction Project (2017ZZ02014); Shanghai Key Laboratory of Emergency Prevention, Diagnosis and Treatment of Respiratory Infectious Diseases (20dz2261100); and Cultivation Project of Shanghai Major Infectious Disease Research Base (20dz2210500).

## Conflict of Interest

The authors declare that the research was conducted in the absence of any commercial or financial relationships that could be construed as a potential conflict of interest.

## Publisher’s Note

All claims expressed in this article are solely those of the authors and do not necessarily represent those of their affiliated organizations, or those of the publisher, the editors and the reviewers. Any product that may be evaluated in this article, or claim that may be made by its manufacturer, is not guaranteed or endorsed by the publisher.
